# Effects of inhalable gene transfection as a novel gene therapy for non-small cell lung cancer and malignant pleural mesothelioma

**DOI:** 10.1038/s41598-022-12624-4

**Published:** 2022-05-23

**Authors:** Misa Ichikawa, Naomi Muramatsu, Wataru Matsunaga, Takahiro Ishikawa, Tomoyuki Okuda, Hirokazu Okamoto, Akinobu Gotoh

**Affiliations:** 1 Institute for Advanced Medical Sciences, Hyogo College of Medicine, 1-1 Mukogawa-cho, Nishinomiya, Japan; 2 StateArt Inc., 2-9-12 Horidome-cho, Nihonbashi, Chuo-ku, Tokyo, Japan; 3 Department of Drug Delivery Research, Faculty of Pharmacy, Meijo University, 150 Yagotoyama, Tempaku-ku, Nagoya, Japan; 4 Joint-Use Research Facilities, Hyogo College of Medicine, 1-1 Mukogawa-cho, Nishinomiya, Japan; 5 Department of Education for Medical Research Base, Hyogo College of Medicine, 1-1 Mukogawa-cho, Nishinomiya, 663-8501 Japan

**Keywords:** Cancer, Drug discovery

## Abstract

Gene therapy using vectors has attracted attention in recent years for the treatment of cancers caused by gene mutations. Besides, new treatments are imperative for lung cancer, including non-small cell lung cancer (NSCLC) and malignant pleural mesothelioma (MPM), due to its high mortality. We developed a minimally invasive and orally inhalable tumor suppressor gene drug (SFD-p16 and SFD-p53) with non-viral vectors for lung cancer treatment by combining tumor suppressor genes with an inhalant powder that can deliver active ingredients directly to the lung. We used NSCLC (A549 and H1299) and MPM (H2052) cell lines in an air–liquid interface culture. Transfection of A549 and H2052 cells with SFD-p16 significantly increased p16 mRNA expression levels and decreased cell proliferation in both cell lines. Similar results were obtained with transfection of H1299 with the inhalable gene drug SFD-p53. In an in vivo experiment, a mouse model of lung cancer with orthotopically transplanted luciferase-expressing A549 cells was subjected to intratracheal insufflation of SFD-p16. Consequently, SFD-p16 effectively and directly affected lung cancer. This study suggests that inhalable gene drugs are effective treatments for NSCLC and MPM. We expect inhalable gene drugs to present a novel gene therapy agent for lung cancer that patients can self-administer.

## Introduction

Lung cancer is one of the most common and serious cancers worldwide, and non-small cell lung cancer (NSCLC) accounts for approximately 85% of all lung cancers^1^. In recent years, advances in targeted therapies such as molecular-targeted drugs and monoclonal antibodies have greatly improved NSCLC treatment^2,3^. The overall survival rate of NSCLC is improving but remains poor in patients with advanced cancer^4^. In Japan, the number of deaths due to asbestos-induced malignant pleural mesothelioma (MPM) is increasing annually. Although the use of asbestos has been prohibited since 2006, it is considered that the number of patients will peak around 2030 because the incubation period after initial asbestos exposure is 25–50 years^5,6^. MPM is an intractable cancer with limited response to surgery and radiation therapy, and the response rate of standard chemotherapy using cisplatin and pemetrexed is approximately 40%. Therefore, new treatment modalities for MPM and NSCLC needs to be researched^7,8^.

Many recent reports have claimed that gene therapy is a new therapeutic modality for NSCLC and MPM^9–11^. As of 2021, over 3,100 clinical studies on gene therapy have been conducted worldwide, 67.4% of which have targeted cancer^12^. Gene delivery techniques often use viral vectors, and we previously reported that the transduction of tumor suppressor genes using viral vectors in various cancer cells inhibits tumor cell proliferation^13^. However, there are significant legal and ethical hurdles and facility requirements to be addressed before gene therapy can be used in Japan, and it is extremely difficult to obtain approval for gene therapy under the current Japanese regulations. Therefore, we have developed a novel non-viral vector that is less invasive and easier to handle than viral vectors to make non-viral vectors preferable for clinical applications.

We directed our attention toward inhalable dry powder particles capable of local drug delivery to the lungs in a direct and noninvasive manner. The particles are challenging to design because the optimal aerodynamic diameter range of aerosol particles for deep lung delivery is 1–5 µm^14^. We reported gene transduction using optimally sized dry powder inhalers for dispersal during inhalation. We observed luciferase gene expression in the lungs of mice that were administered an oral spray of a powdered preparation combining the luciferase gene and hyaluronic acid (HA) as an excipient^15^. Scanning electron microscope (SEM) observation and Andersen cascade impactor (ACI) evaluation confirmed that the powdered gene preparation combining HA had a specific hollow porous particle shape and an aerodynamic diameter suitable for deep lung delivery. We combined this dry powder inhaler technology using HA with a tumor suppression gene to develop a new gene drug for treating NSCLC and MPM. We expect that such a gene drug will be a suitable therapeutic agent for lung cancer that patients can inhale themselves.

In this study, we used inhalable gene drugs, spray freeze-dried (SFD)-p16 or SFD-p53, which contain the tumor suppressor genes *p16*^*INK4a*^ or *p53*, respectively, to evaluate gene expression after transfection in NSCLC (A549 and H1299) and MPM (H2052) cell lines, as frequent mutations of *p16*^*INK4a*^ and *p53* have been reported in MPM and NSCLC^16–19^. We also report their inhibitory effects on tumor cell proliferation in vitro and in vivo to validate their efficacy in NSCLC or MPM treatment.

## Results

### In vitro gene expression and inhibitory growth effect of inhalable gene drugs

**Figure 1:**
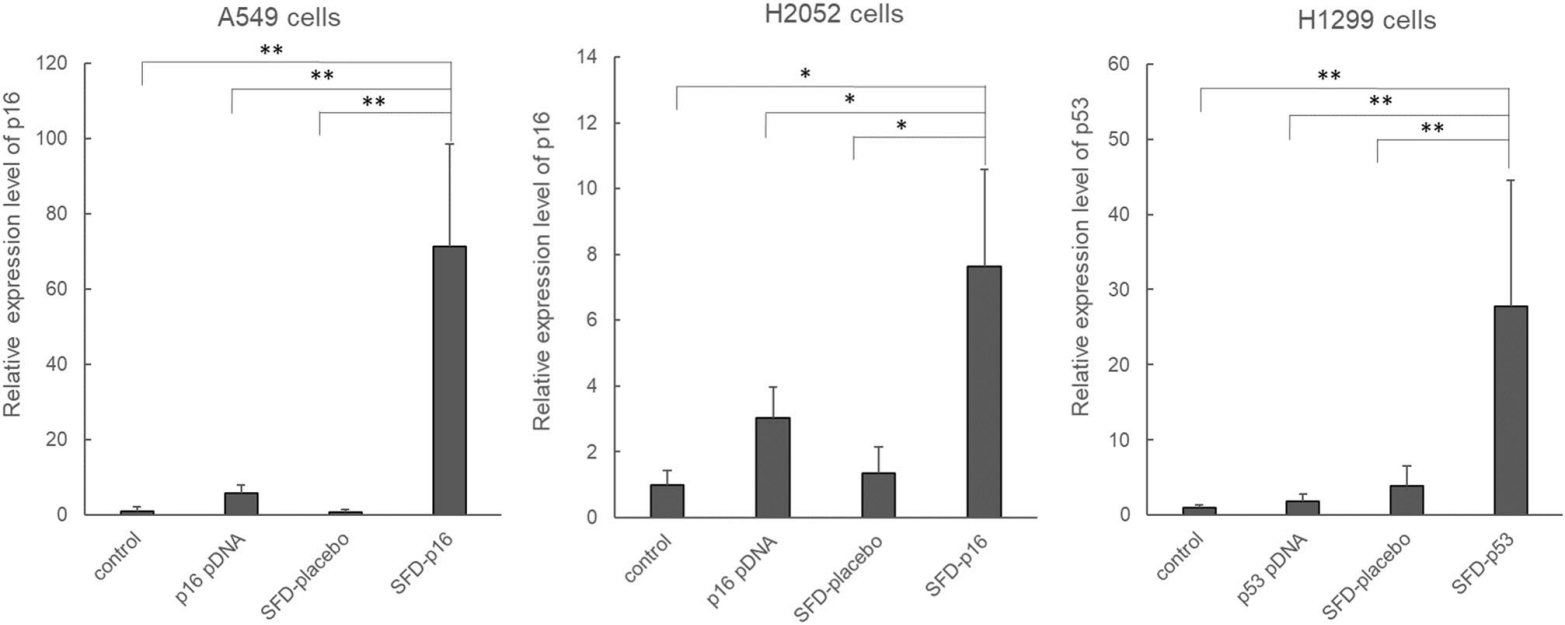
Real-time PCR analysis of gene expression in each cell line 24 h after inhalable gene transfection. In A549 and H2052 cells, the p16 expression of SFD-p16 was significantly higher than that in the control (N = 3). In H1299 cells, the p53 expression of SFD-p53 was significantly higher than that in the control (N = 6–7). The results were analyzed using a one-way ANOVA followed by Bonferroni multiple comparisons tests and Student’s t-test. The data represents the means ± SD (1 = gene expression level of control). **p < 0.01, *p < 0.05.

To investigate the gene expression of inhalable gene drugs, we analyzed the relative gene expression levels using real-time PCR 24 h after of transfection in cells cultured by the ALI culture method (Fig. 1). This experiment was performed in four groups: control, pDNA, SFD-placebo, and SFD-p16 or SFD-p53. We confirmed expression levels of p16 mRNA in A549 and H2052 cells and that of p53 mRNA in H1299 cells.

In A549 and H2052 cells, the p16 expression of SFD-p16 was significantly higher than that in the control (A549, f_3,8_ = 12.919, *p* < 0.01; H2052, f_3,8_ = 7,214, *p* = 0.012). In H1299 cells, the p53 expression of SFD-p53 was significantly higher than that in the control (f_3,21_ = 11.394, *p* < 0.01). p53 pDNA did not exhibit any significant expression.

**Figure 2:**
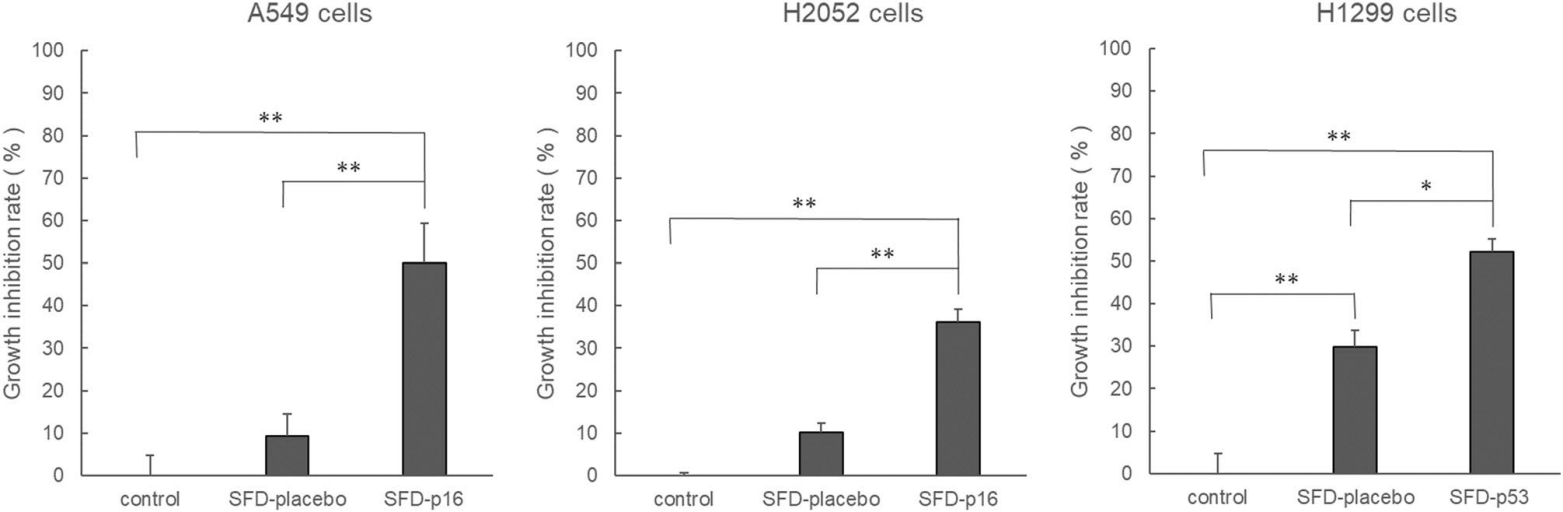
Cell growth inhibition rate of each cell line 24 h after inhalable gene transfection. Significant growth inhibition by inhalable gene drugs (SFD-p16 or SFD-p53) was observed in all cell lines. The results were analyzed using a one-way ANOVA followed by Bonferroni multiple comparisons tests. The data represents the means ± SD of the percentage (0% = cell inhibition rate of control). N = 3, **p < 0.01, *p < 0.05.

At the same time, we counted viable cells in three groups (control, SFD-placebo and SFD-p16 or SFD-p53) of each cell line. Figure 2 shows the cell growth inhibition rate of SFD-placebo and SFD-p16 or SFD-p53 for A549, H2052, and H1299 cells. In A549 cells, a one-way ANOVA detected significant between-group differences (f_2,6_ = 30.620, *p* < 0.01). The growth inhibition rate of SFD-p16 (49.9%) was significantly higher than that of the control and SFD-placebo groups (9.3%). SFD-placebo did not exhibit a significant effect. Similarly, a one-way ANOVA detected significant between-group differences (f_2,6_ = 19.406, *p* < 0.01) in H2052 cells. The growth inhibition rate of SFD-p16 (36.1%) was significantly higher than that of the control and SFD-placebo groups (10.1%). SFD-placebo did not exhibit a significant effect. In H1299 cells, a one-way ANOVA detected significant between-group differences (f_2,6_ = 27.464, *p* < 0.01). SFD-placebo (29.8%) and SFD-p53 (52.1%) significantly suppressed cell growth, but SFD-p53 was significantly more effective than SFD-placebo.

### Efficacy of SFD-p16 in nude mice with subcutaneous transplantation of lung cancer A549 cells ex vivo and in vivo

#### Ex vivo* test*

**Figure 3:**
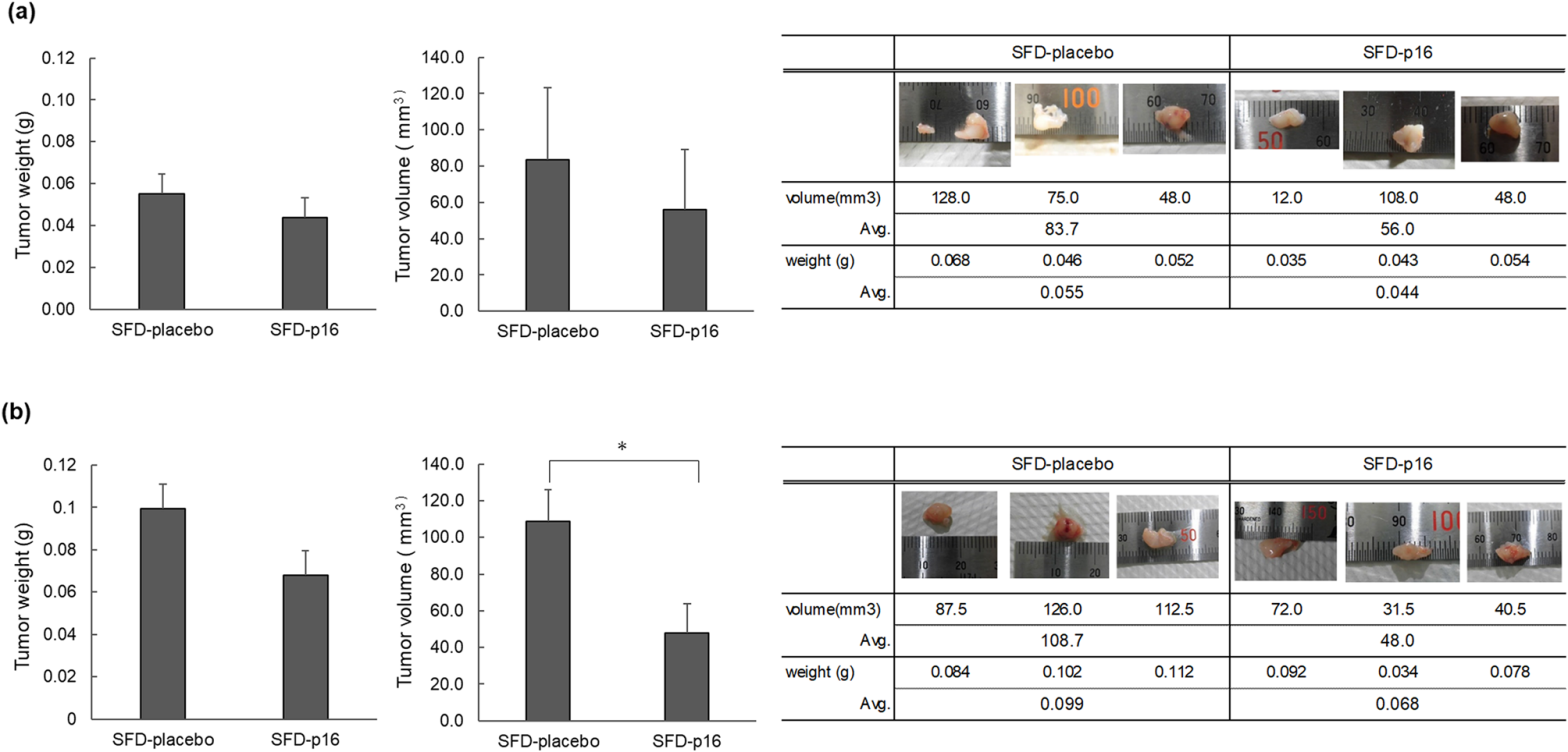
Tumor weight and volume in nude mice with subcutaneous transplantation of A549 cells ex vivo and in vivo after SFD powder treatments. (**a**) Ex vivo test. After treating A549 cells with SFD-p16 or SFD-placebo, subcutaneous transplantation was performed. One week later, tumor weight and volume were measured and compared. No significant difference was observed; however, tumors in the SFD-p16 group tended to be lighter. The data represents the means ± SD (N = 3). Mean values were compared using a one-tailed Student’s t-test. (**b**) In vivo test. Tumors formed after subcutaneous transplantation were treated with SFD-p16 and SFD-placebo. One week after treatment, the tumor weight and volume were measured and compared. Tumors in the SFD-p16 group were significantly smaller. The data represents the means ± SD (N = 3). *p < 0.05. Mean values were compared using a one-tailed Student’s t-test. Avg.: Average.

A549 cells sprayed with SFD-p16 or SFD-placebo in advance were subcutaneously transplanted into nude mice, and the effect on tumorigenesis was confirmed. One week later, the tumors were removed, and their weights and sizes were measured. As shown in Fig. 3a, there was no significant difference between SFD-p16 and SFD-placebo; however, SFD-p16 tended to suppress tumor growth.

#### In vivo* test*

To investigate the effect of the dry gene powder on tumors, SFD-p16 and SFD-placebo were sprayed on subcutaneously generated tumors in nude mice, and the tumor weight and size were measured one week later. The average volume of the tumor in the SFD-p16 group was significantly smaller than that of the SFD-placebo group (*p* = 0.021; Fig. 3b).

### Tumor-suppressive effects of SFD-p16 using A549 cells in orthotopic lung cancer model in vivo test

**Figure 4:**
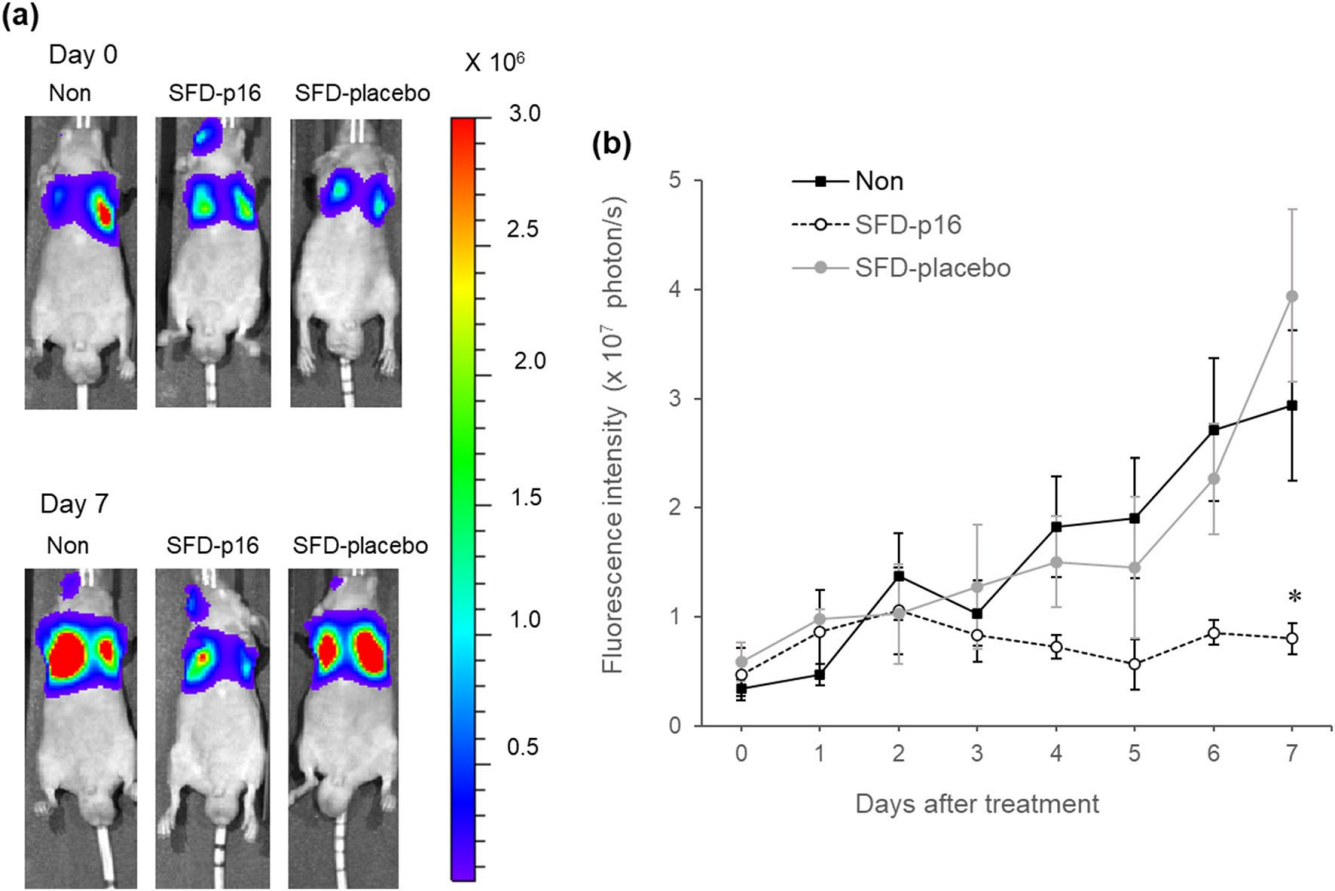
Tumor-suppressive effects of SFD-p16 in the orthotopic lung cancer model in vivo test. (**a**) Optical images of lung luminescence corresponding to firefly luciferase activity in mice bearing A549/Luc cells with no treatment or treated with SFD-p16 or SFD-placebo. A549/Luc cells were intravenously injected into the retro-orbital venous sinus at day 0. The luminescence corresponding to firefly luciferase activity was detected using IVIS. SFD-p16 and SFD-placebo were intratracheally insufflated at a dose of 0.5 mg/mouse. The color scales are in photons/s/cm^2^/sr. (**b**) Time course of lung luminescence intensity corresponding to firefly luciferase activity in mice bearing A549/Luc cells with no treatment (the solid line) or treated with SFD-p16 (the dotted line) or SFD-placebo (the gray line). Each value represents the mean ± SE (n = 5–12). Homogeneity of variance was determined by Levene's test. Between-group statistical differences were compared using Dunnett's test. (*p < 0.05 compared with Non). Non: no treatment; SE: standard error.

To verify the anti-tumor effects of SFD-p16 on the lung through inhalation delivery, BALB/c *nu/nu* mice bearing A549/Luc cells were imaged in vivo (Fig. 4). On day 4 after treatment, the luminescence in the lungs of the control mice continued to increase, indicating tumor growth. On the other hand, intratracheal administration of SFD-p16 inhibited the increase in luminescence in the lungs compared to no treatment, while the SFD-placebo treatment did not. The suppression of luminescence by SFD-p16 treatment was significant on day 7. These results strongly indicate that SFD-p16 effectively repressed the growth of tumor cells in the lungs of mice.

## Discussion

This study aimed to develop gene therapy for the efficient and effective treatment of cancer using non-viral vectors. In addition, the currently available genetic drug is invasive, requiring intravenous or local administration; thus, we sought an administration method that could reduce the burden on patients as much as possible. Clinically employed non-viral vectors include cationic liposomes; however, the efficiency of gene transfer into tumors is a challenge in the case of systemic administration^20,21^. To address such challenges, we developed an orally inhalable gene drug by combining tumor suppressor genes with an inhalant powder that can deliver active ingredients directly to the lung in a noninvasive manner. We previously reported that inhalable gene drugs with HA as an excipient showed higher gene expression and dispersion in the lungs than polyethyleneimine (PEI) known as a common gene transfer agent^15^. By performing both in vitro and in vivo experiments, we demonstrated that inhalable gene drugs containing tumor suppressor genes were more effective in suppressing the proliferation of NSCLC and MPM than a placebo dry powder that did not contain tumor suppressor genes (SFD-placebo).

Various gene mutations have been reported in patients with NSCLC and MPM. Given the high frequency of *p16*^*INK4a*^ and *p53* mutations among these reported mutations, we prepared inhalable gene drugs for these two genes (SFD-p16 and SFD-p53, respectively)^22,23^. SFD-p16 was used for NSCLC A549 and MPM H2052 cells, in which p16 deletion has been reported^24,25^. In contrast, SFD-p53 was used in NSCLC H1299 cells, which lack p53^26^. After spraying SFD-p16 or SFD-p53 onto A549, H1299, and H2052 cells in ALI culture for 24 h, the expression of the *p16*^*INK4a*^ or *p53* gene was confirmed by real-time PCR. Consequently, it was ascertained that these inhalable gene drugs had high gene transfer efficiency, with the cells exhibiting high expression rates. Meanwhile, gene expression levels were low with naked pDNA.

This may be because DNA is negatively charged and, hence, is repelled by negatively charged cell surfaces. Therefore, the transfection of pDNA alone is not very efficient^27^. Curiously, HA is also incorporated into cells despite its negative charge^28^; thus, we speculate that inhalable gene drugs are taken up by cells via the HA receptor. Previous reports have demonstrated that both NSCLC and MPM overexpress the HA receptor CD44^29,30^. Therefore, we hypothesize the following: first, the inhalable gene drugs are dispersed, and the contained HA binds to CD44 on the cell surface. Second, transgenes are taken up by cells via endocytosis.

This experiment did not reveal the mechanism of gene transfer by inhalable gene drugs, and we plan to elucidate the underlying mechanism by conducting further basic research in the future. In all cells subjected to the inhalable gene drugs, cell proliferation was significantly suppressed 24 h after transfection compared with the control and SFD-placebo groups. We believe that this result reflects the suppressive effects of tumor suppressor genes on cell proliferation. In contrast, SFD-placebo showed limited but significant inhibition of cell growth in H1299 cells. It cannot be ruled out that HA itself may have affected cell growth, but there was no significant tumor inhibitory effect of SFD-placebo in vitro and in vivo using other cells. The cell difference of the sensitivity to HA might be related to this result. Thus, we will proceed with research for elucidation in the future considering the differences based on the cell type, if applicable. Although a recent study reported that high-molecular weight HA suppresses tumorigenicity and low-molecular weight HA is oncogenic^31^, we consider 50 kDa HA was safe because it did not promote cell proliferation in this experiment.

The ex vivo treatment of cells sprayed with SFD powder was first performed in the subcutaneous transplant animal experiment. The ex vivo treatment with SFD-p16 showed no significant effect but tended to suppress tumor growth. The SFD powder cannot maintain its properties when dissolved^15^. Therefore, gene transfer into the cell might not be sufficient because the SFD powder was dissolved in the medium that could not be completely removed when it was sprayed. Thus, we hypothesized that it would be easier to observe the drug’s effects after the subcutaneously transplanted tumor had grown; we, thus, conducted in vivo experiments. As a result, the volume of tumors sprayed with SFD-p16 was significantly smaller than those sprayed with SFD-placebo.

In our previous study, we have observed luciferase gene expression in the lungs of mice that were administered an oral spray of a powdered preparation combining the luciferase gene and HA as an excipient^15^. Moreover, the aerodynamic diameter of SFD-p16 was measured using an Andersen cascade impactor (ACI), and it was confirmed that the particles can reach deep into the lungs (No data). In the in vivo experiment, the mouse model of lung cancer with orthotopically transplanted A549/Luc cells was subjected to intratracheal insufflation of SFD-p16. Seven days after treatment, the SFD-p16 group showed a significant difference in the cancer cells' luminescence intensity compared to that in the other treatment groups. This confirmed the suppressive effects of the tumor suppressor genes of SFD-p16 on cell proliferation. In other words, the orally inhalable gene drug SFD-p16 effectively and directly affects lung cancer.

These findings suggest that cancer gene therapy using inhalable gene drugs may constitute a novel therapeutic regimen for NSCLS and MPM and improve patients' quality of life owing to the low invasiveness of the mode of administration.

Although research on novel cancer therapies is advancing each day, the high global mortality rate due to lung cancer remains a serious challenge. NSCLC accounts for approximately 85% of all lung cancers, and the number of new tracheal, bronchus, and lung cancer cases increased by 23.3% globally from 2010 to 2019^32^. MPM is extremely refractory to conventional treatment modalities, and given the prediction that the incidence of MPM will peak around 2030 in Japan, it is feared that the number of patients will continue to grow. According to the Global Burden of Disease database, US, UK, and China are the top three countries in terms of the incidence of mesothelioma. While the incidence is almost at its peak in western nations, the number of patients is expected to increase further in China, the largest consumer of asbestos globally^33^. Amid such circumstances, minimally invasive and easy-to-administer inhalable gene drugs are expected to contribute greatly to the treatment of NSCLS and MPM.

## Methods

### Inhalable gene drugs

In this study, we prepared three SFD powders in the same manner as previously reported^15^. Briefly, the solution composed of the following ingredients was rapidly frozen by spraying them at 150 kPa and 5 mL/min into liquid nitrogen from the nozzle tip of a spray dryer to generate frozen droplets. The frozen droplets were freeze-dried under vacuum for 24 h to obtain the desired SFD powders. The powders were constructed with 50 kDa HA (Kikkoman Biochemifa Co., Tokyo, Japan), L-phenylalanine (Sigma-Aldrich, St. Louis, MO) as a dispersion agent, and plasmid DNA (pDNA; Table 1). We used two different tumor suppressor genes that code human *p16*^*INK4a*^ (SFD-p16) and human *p53* (SFD-p53), driven by the cytomegalovirus promoter, containing 10 µg in 0.5 mg of powder. The powder without pDNA was prepared as an SFD placebo, and 1% of indocyanine green (ICG; Fujifilm Wako Pure Chemical Co., Osaka, Japan) was added as a fluorescent label for the dry powders in the orthotopic lung cancer model in vivo.

**Table 1:** Composition of Dry powders (%).

	pDNA	HA	Phe
SFD-placebo	0	75	25
SFD-p16	2	73	25
SFD-p53	2	73	25

Phe; L-phenylalanine.

### Cell lines and cell culture

The human NSCLC cell line A549 (*p16*^*INK4a*^- homozygous deletion), H1299 cells (*p53*- homozygous partial deletion), and the human MPM cell line H2052 (*p16*^*INK4a*^—homozygous deletion) were purchased from the American Type Culture Collection (Manassas, VA, USA). The cells were cultured in Roswell Park Memorial Institute 1640 (RPMI1640) supplemented with 10% fetal bovine serum (FBS) and 100 U/mL penicillin–streptomycin and incubated in 5% CO_2_ at 37 °C. In the orthotopic lung cancer model in vivo, A549 cells expressing luciferase (A549/Luc), purchased from the Japanese Collection of Research Bioresources Cell Bank (Osaka, Japan), were cultured under the same conditions as above.

### Air–liquid interface (ALI) and cell counting

ALI culture was performed in three cell lines. 2.0 × 10^5^ cells from each cell line were seeded into the insert of a Transwell 24-well plate (Corning Inc., Corning, NY) and 1 mL of the RPMI1640 culture medium was added. After culturing for 48 h, the medium in each well was replaced with fresh Opti-MEM or RPMI1640, and the insert previous medium was removed. We sprayed 0.5 mg SFD-placebo and SFD-p16 or SFD-p53 onto the cell surface using an administration device prepared by connecting a 1 mL syringe (Terumo Co., Tokyo, Japan) with a three-way stopcock (Top Co., Tokyo, Japan) and subsequently incubated them with ALI for 24 h. Details of the administration method have been previously reported^15^. Untreated cells were used as the negative controls. The adherent cells were collected with trypsin/EDTA. These were counted using a hemocytometer following staining with 0.4% trypan blue. The cell growth inhibition rate was calculated as a percentage using the following formula: 100-Ax/An × 100 (where An = viable cell count of control and Ax = viable cell count of dry powders).

### Real-time polymerase chain reaction (PCR)

After 24 h of transfection in four groups, control, pDNA, SFD-placebo, and SFD-p16 or SFD-p53, the cells cultured by ALI were collected. The pDNA group was 10 µg of naked plasmid DNA of p16 or p53 in phosphate-buffered saline (PBS). The collected cells were lysed with 250 µL PBS and 750 µL RNAiso Blood (Takara Bio, Otsu, Japan), and RNA was extracted according to the manufacturer's protocol. Reverse transcription-polymerase chain reaction (RT-PCR) and qualitative RT-PCR were carried out according to the manufacturer’s instructions for the Thunderbird SYBR qPCR/RT Set (Toyobo, Osaka, Japan) in a StepOnePlus™ Real-Time PCR System (Applied Biosystems, Foster City, CA, USA). The reaction program was initiated at 95 °C for 20 s, followed by 40 cycles of denaturation at 95 °C for 5 s, and annealing and extension at 60 °C for 20 s; and the melting curve was determined at 95 °C for 15 s, 60 °C for 1 min, and 95 °C for 15 s. The primer sequences used are listed in Table 2.

**Table 2:** Qualitative real-time PCR primer nucleotide sequences.

Cell	Primer	Forward	Reverse
A549	p16^INK4a^ -1	5'-CCCCTTGCCTGGAAAGATAC-3'	5'-AGCCCCTCCTCTTTCTTCCT-3'
	HPRT1	5'-AGATGGTCAAGGTCGCAAG-3'	5'-GTATTCATTATAGTCAAGGGCATATCC-3'
H2052	p16^INK4a^ -2	5'- CTGTCCTGCGTGTTGAAAGA-3'	5'- TTGGGTAATTTTTGGGATCTACA-3'
	PPIA	5′-GAGGAAAACCGTGTACTATTAGC-3′	5′-GGGACCTTGTCTGCAAAC-3′
H1299	p53	5'-CCAGGGCAGCTACGGTTTC-3'	5'-CTCCGTCATGTGCTGTGACTG-3'
	PPIA	5′-GAGGAAAACCGTGTACTATTAGC-3′	5′-GGGACCTTGTCTGCAAAC-3′

For primer information, refer to the following references^37–41^. The primers were selected as appropriate during the actual analysis.

### Ex vivo treatment in nude mice with lung cancer cell subcutaneous transplantation

The cultured A549 cells (2.5 × 10^7^) were collected and sprinkled with about 0.5 mg each of SFD-p16 or SFD-placebo after the medium was removed sufficiently. Approximately 30 min later, the cells (2.5 × 10^7^) were mixed with 50 μL of Matrigel and were transplanted subcutaneously into the back of BALB/c *nu/nu* nude mice (5-week-old males) purchased from CLEA Japan, Inc. (Tokyo, Japan). One week later, the tumor was removed and fixed with 4% paraformaldehyde (PFA) for 3 days before measuring the weight and size of the tumor. Then, we calculated the tumor volume (mm^3^) as (width^2^ × length)/2. Animal experiments with subcutaneously transplanted mice were performed per the animal experiment regulations of the Hyogo College of Medicine and the ARRIVE guidelines.

### In vivo treatment in nude mice with lung cancer cell subcutaneous transplantation

The cultured A549 cells (2.5 × 10^7^) mixed with 50 μL of Matrigel were injected subcutaneously into the back of BALB/c *nu/nu* mice (5-week-old males). After confirming tumor formation 1 week later, the mice’s skin was incised and about 0.5 mg of SFD-p16 and SFD-placebo was sprayed onto the tumor by releasing 0.25 mL of air compressed in a syringe. The skin was then sutured. One more week later, the tumor was removed and fixed with 4% PFA for 3 days before measuring the weight and size of the tumor. Then, we calculated the tumor volume (mm^3^) as (width^2^ × length)/2.

### Oral inhalation treatment of inhalable gene drug in orthotopic transplant mice

The animal experiments on orthotopically transplanted lung cancer mice were approved by the animal experiment and welfare committee at Meijyo University and performed following the Guiding Principles for the Care and Use of Laboratory Animals approved by the Faculty of Pharmacy, Meijo University. This study was also carried out in compliance with the ARRIVE guidelines. BALB/c *nu/nu* mice (6-week-old males) were purchased from Japan SLC (Hamamatsu, Japan).

Each mouse was anesthetized with isoflurane during the injection of A549/Luc cells (5.0 × 10^6^) through the retro-orbital venous sinus. After the luminescence intensity, determined using an in vivo imaging system (IVIS; IVIS-SPECTRUM, Caliper Life Sciences, Hopkinton, MA, USA), in the lungs reached 5.0 × 10^5^ photons/s (on day 4 following inoculation), the treatments were started.

The administration of inhalation powder, SFD-p16 or SFD-placebo, was performed as previously described^34^. Briefly, on day 4 after A549/Luc inoculation, each mouse was anesthetized with a mixture of three anesthetics (0.3 mg/kg body weight [BW] medetomidine, 4.0 mg/kg BW midazolam, and 5.0 mg/kg BW butorphanol), and a 4 cm PE-60 polyethylene cannula was inserted into the trachea via the mouth. The administration device’s tip was inserted into the cannula, and the powder was dispersed at a dose of 0.5 mg/mouse by releasing 0.25 mL of air compressed in a syringe. Another group of mice was left untreated throughout the experiment.

Following pulmonary administration, fluorescence and luminescence were detected using IVIS, as described in our previous reports^35,36^. ICG fluorescence was observed to confirm the gene powder’s successful distribution in the lungs. To detect the luminescence corresponding to luciferase activity, luciferin as a substrate of luciferase was intranasally administered (75 mg/kg BW) 10 min before the detection point, and the exposure time was set to 1 min.

To quantify the luminescence intensities in the lungs of mice, the region of interest was adjusted to a rectangle with a width of 3 cm and a height of 1 cm. The luminescence intensity (total flux [photon/s]) was determined as A549/Luc cell growth.

### Statistical analysis

Statistical analyses were performed using JSTAT ver.22.0 J software and BellCurve for Excel ver. 3.21. The results are expressed as the mean ± standard deviation (SD) unless otherwise indicated. Mean values were compared using a one-way analysis of variance (ANOVA) followed by Bonferroni or Dunnett multiple comparisons tests and a one-tailed Student’s t-test, as indicated in the figure legends. The significance levels were set at *p* < 0.05 and *p* < 0.01.
